# Supplementary material for: Comparative Genomics of Beggiatoa leptomitoformis Strains D-401 and D-402T with Contrasting Physiology But Extremely High Level of Genomic Identity
Source: Microorganisms. 2020 Jun 19;8(6):928. doi: 10.3390/microorganisms8060928 (PMC7356093; doi:10.3390/microorganisms8060928)
Supplement: Supplementary file 1 [file microorganisms-08-00928-s001.pdf]

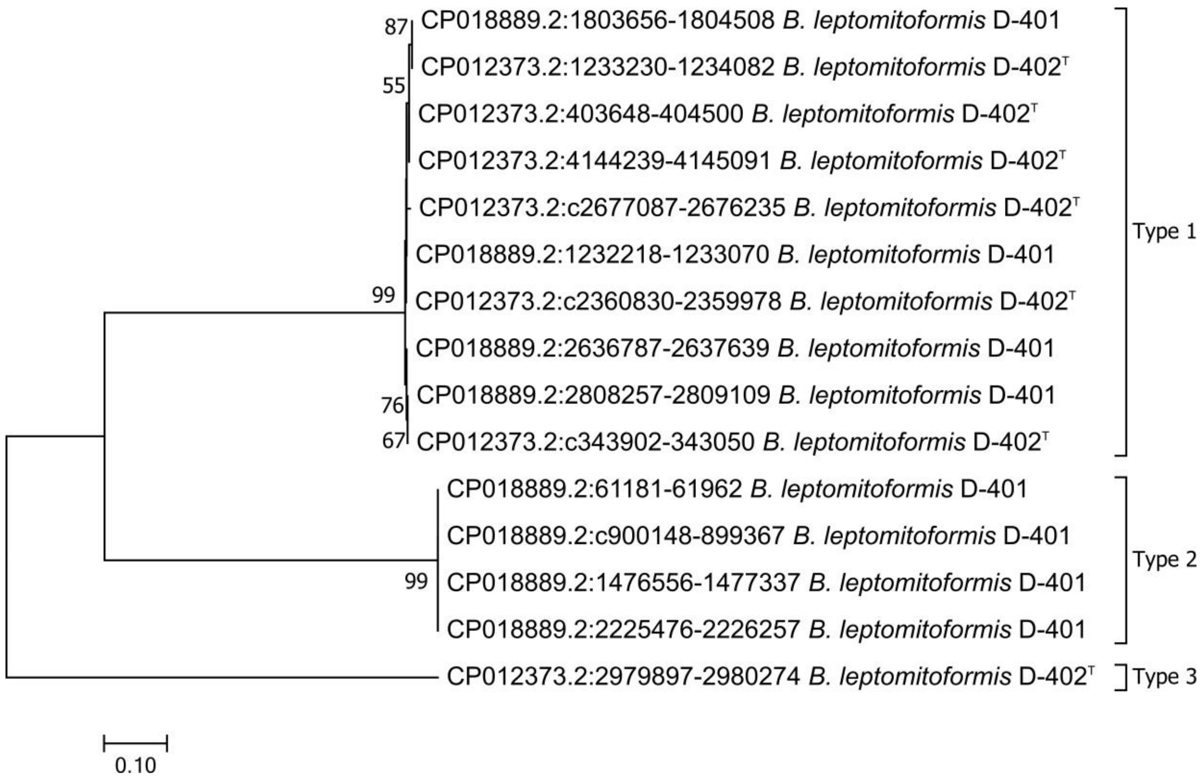

Figure S1. Dendrogram of 15 long inserts found in the genomes of *B. leptomitoformis* strains D-401 and D-402^{T}.

Table S1. Inserts of *B. leptomitoformis* strain D-401.

Coordinate of insert start in D-401 (bp)	Coordinate of insert end in D-401 (bp)	Coordinate of insert in D-402^{T} (bp)	Insert length (bp)
61181	61962	61181	782
899367	900148	900303	782
1232218	1233070	1232372	853
1476556	1477337	1476710	782
1803656	1804508	1803028	853
2225476	2226257	2223995	782
2636787	2637639	2635377	853
2808257	2809109	2806848	853
3201146	3201146	3199262	1
3202340	3202340	3200455	1

Table S2. Inserts of *B. leptomitoformis* strain D-402^T.

Coordinate of insert start in D-402^T (bp)	Coordinate of insert end in D-402^T (bp)	Coordinate of insert in D-401 (bp)	Insert length (bp)
343050	343902	343832	853
344559	344569	344488	11
403648	404500	403566	853
898872	898872	897937	1
1233230	1234082	1233929	853
2359978	2360830	2362241	853
2676235	2677087	2678498	853
2773108	2773108	2774518	1
2979897	2980274	2982159	378
4144239	4145091	4146125	853

Table S3. Inverted and direct repeats at the ends of long inserts.

Transposon	Inverted repeat	Direct repeat
Type 1	grgcstgtcatcaaata	ta
Type 2	ggtagtgctaaaga	n_8

Table S4. Genes in a full-length transposon type 1 sequence.

D-401 Locus tag	D-402^T Locus tag	Protein
BLE401_00700	AL038_05285	IS5 family transposase
BLE401_00695	AL038_05290	IS630 family transposase
BLE401_00690	AL038_05295	transposase
BLE401_00685	AL038_05300	transposase

Table S5. Genes in a full-length transposon type 2 sequence.

D-401 Locus tag	D-402^T Locus tag	Protein
BLE401_08135	AL038_16115	hypothetical protein
BLE401_08130	AL038_16120	IS630 family transposase
BLE401_08125	AL038_16125	transposase
BLE401_08120	AL038_16130	IS1 family transposase

Beggiatoa leptomitoformis D-402^T (ALG67575.1)

MLKIAKVLALSTVLGAGFVAAPAHAWWGGPGSGWGNNEWGPFDGSGWGDFNMSMGGGGRGYGRQ
YNNYNYSGYPGYYGGYAPAPAYGAAPYGGYAPAPYAGYGVAPAPYAGYGAAPYGGGYAPAPYAG
YGVAPYGAVPPASVPAPAAPEAK

Beggiatoa alba B18LD (EIJ43877.1)

MLKIAKVLALSAVLGAGVAAAPAQAWWGAPGWGPGSGSGWNDWGPFDGSGWGDFNMSMGGGGRG
YGRQYSNYGYYPYGGYGGYPYGGGYGYPYGGYAPATPYGYAPAAPYGYAPPYGAVPAIPPTAPT
APAPDAK

Thioflexithrix psekupsensis D3^T (OUD13142.1)

MKMTAKALAISTILGAAALAATPAQAWWGWGPGSGWGGPWGGGPGSSAYDIGPFDGSGWGDFNM
SMGGGGRGYGSGRNHYQGYGYPVYGYGYPVYGYGYPGYGYGYPAPAYGYPGYGAVPPVPPPPAY
GVAPEYSAPAAPEKAK

Figure S2. The protein sequences which are supposed to be a sulfur globule protein. N-terminal transport peptides are marked blue; prolines are marked yellow.

Table S6. Identification results of *B. Leptomitoformis* proteins by MALDI mass spectrometry.

Protein	RatedMw, kDa	MS/MS identifiedpeptides in the spot
ALG67575.1	15.071	GGPGSGWGNNEWGPFDGSGWG DFNMSMGGGGR
		PAPYAGYGVAPYGAVPPASVPA
ALG68134.1	47.384	NFDGLDRDD
		GDKYSSYGFGLVQA

A.

MSFSKHLQVGCASALVALAPFAMADDAADMKR**QLQQLQQRLDTLESNPGMSLGAKPKFSLQLSG**
QVNRAVMYGDDGTDSDTFFVDGDTDASRVEILGKHSLSDDVTLGTNIEVE**YLSNSSSEVDINQT**
KSVDHNGFSERTLEFYAESATLGRLSLGQGSMASNDTAEYDLNSSSLAGMY**SFGADFGANLSFR**
DADTKAK**IATIGQVHSNFDGLDRDDRIRYDTPSFGGLTLSVAASTAEKWDAGITFQR**TFGPIDL
ALGLGYGNVGDSEAYLYEDDEGNTYQLYK**HVYSASFSILHTPSGLSMTLAGGGLSNDSLTTTIK**
DLPSNVSDTLGDNREDTDPFSWSAKLGYAIDATSIGKTSFGLSYNRTEDYR**TCLGYDSVNNTDV**
FLSCGDKYSSYGFGLVQAIDKASTEIYFGARNHQLESAGLNPDDILTIYSGARVKF

B.

MLKIAKVLALSTVLGAGFVAAPAHAWWGGPGSGWGNNEWGPFDGSGWGDFNMSMGGGGRGYGRQ
YNNYNYSGYPGYYGGYAPAPAYGAAPYGGYAPAPYAGYGVAPAPYAGYGAAPYGGGYAPAPYAG
YGVAPY**GAVPPASVPAPAAPEAK**

Figure S3. Covering the protein sequence of ALG68134.1 (**a**) and ALG67575.1 (**b**) with identified peptides. Peptides which were identified by HPLC-MS/MS are shown in bold; peptide sequences which were identified by MALDI mass spectrometry are underlined.
